# Impact of sentinel pile on botulinum toxin treatment of chronic anal fissure: A comparative study

**DOI:** 10.1007/s10151-026-03309-5

**Published:** 2026-05-01

**Authors:** Javid Ahmadov, Mustafa Anıl Turhan, Ender Ergüder, Sezai Leventoglu, Bülent Mentes

**Affiliations:** 1 Department of Surgery, Faculty of Medicine, Gazi University, Ankara, Turkey; 2 Proctology Unit, Ankara Memorial Hospital, Ankara, Turkey; 3 Department of Surgery, Ankara Etlik City Hospital, Ankara, Turkey

**Keywords:** Chronic anal fissure, Sentinel tag, Sentinel pile, Botulinum toxin

## Abstract

**Aim:**

The aim of this study was to investigate if the presence of a prominent sentinel pile (SP) had any impact on the treatment success of botulinum toxin (BT) injection, as well as the clinical presentation of patients with chronic anal fissure (CAF).

**Methods:**

Patients with CAF with or without prominent sentinel piles underwent BT injection. In addition to objective healing, a detailed symptom severity score (REALISE) immediately before and 6 months after BT injection was recorded. This was a retrospective, single-center observational cohort study including consecutive patients treated in a specialized proctology unit.

**Results:**

Of the 249 patients, 68 presented with prominent SP (27.3%). The overall objective healing rate among all patients who received a single injection of BT was found to be 74.7% at 2 months. When stratified, age distribution was similar between patients with (SP+) and without SP (SP−) (*p* = 0.545). However, SP was more prevalent in female patients (*p* = 0.009). The objective healing rates after a single BT injection were 80.7% in the SP− group and 58.8% in the SP+ group (*p* = 0.001). Pre-treatment REALISE scores were significantly reduced in both groups after BT injection (*p* < 0.001 for both). However, post-treatment scores were higher in the SP+ group compared with the SP− group (*p* < 0.001). Multivariable analysis confirmed SP presence as an independent predictor of reduced objective healing after BT injection.

**Conclusions:**

Even with the SP, a considerable proportion of patients with CAF heal after BT treatment and their symptoms are generally relieved. However, symptomatic improvement is less marked and the objective healing rates are lower in the SP+ group. The presence of SP may, therefore, negatively influence the clinical effectiveness of BT treatment of CAF. These findings should be interpreted in light of the retrospective single-center design and the potential for selection and recall bias.

## Introduction

An anal fissure refers to a tear in the mucosal lining of the distal anal canal. This condition is prevalent, particularly among young, otherwise healthy adults, and affects both sexes [1]. Anal fissure is a common anorectal condition worldwide, accounting for approximately 10–15% of all proctologic outpatient consultations [2]. Population-based epidemiologic data specifically addressing chronic anal fissure are limited; however, in a large population-based cohort, 14.2% of patients with anal fissure were reported to have chronic or recurrent disease [3]. The cardinal symptom of pain is often described as excruciating, akin to the sensation of “passing glass,” while the associated bleeding is typically minimal [4, 5]. Moreover, chronic anal fissure (CAF) significantly disturbs quality of life (QoL) [6–8]. In general, the primary objective of all treatments is aimed at reducing the existing hypertonicity of the sphincter. According to current guidelines and published literature, botulinum toxin (BT) is an established therapeutic option for chronic anal fissure, with efficacy comparable to conservative/medical treatments, and has been shown to provide modestly improved healing rates when used after failure of conservative/medical management [9, 10]. In a previous study, it was reported that BT injection achieved a high healing rate in patients with chronic anal fissure; however, both early and late healing rates were lower than those observed in the sphincterotomy group [11]. BT treatment of CAF has maintained its place in almost all current guidelines [9, 12].

CAF is characterized as a deep, intractable ulcer, the internal anal sphincter (IAS) being visible in its base. Classically, a fissure triad is described—comprising a skin tag (sentinel pile), anal papilla, and the fissure itself—which are also regarded as indicators of poor response to conservative therapy [13–16]. However, the significance of an associated sentinel pile (SP), its pathophysiology, its possible impact on healing of CAF with established treatment methods, or even the prevalence of SP have not been investigated in detail.

The aim of this study was to investigate if the presence of a prominent SP had any impact on the treatment success of BT injection, as well as the clinical presentation of patients with CAF. For detailed clinical evaluation before and after treatment, we utilized the REALISE scoring system, which noted issues, such as the QoL or duration of pain, in addition to the severity of pain [17].

## Patients and methods

### Patient selection and study design

**Fig. 1:**
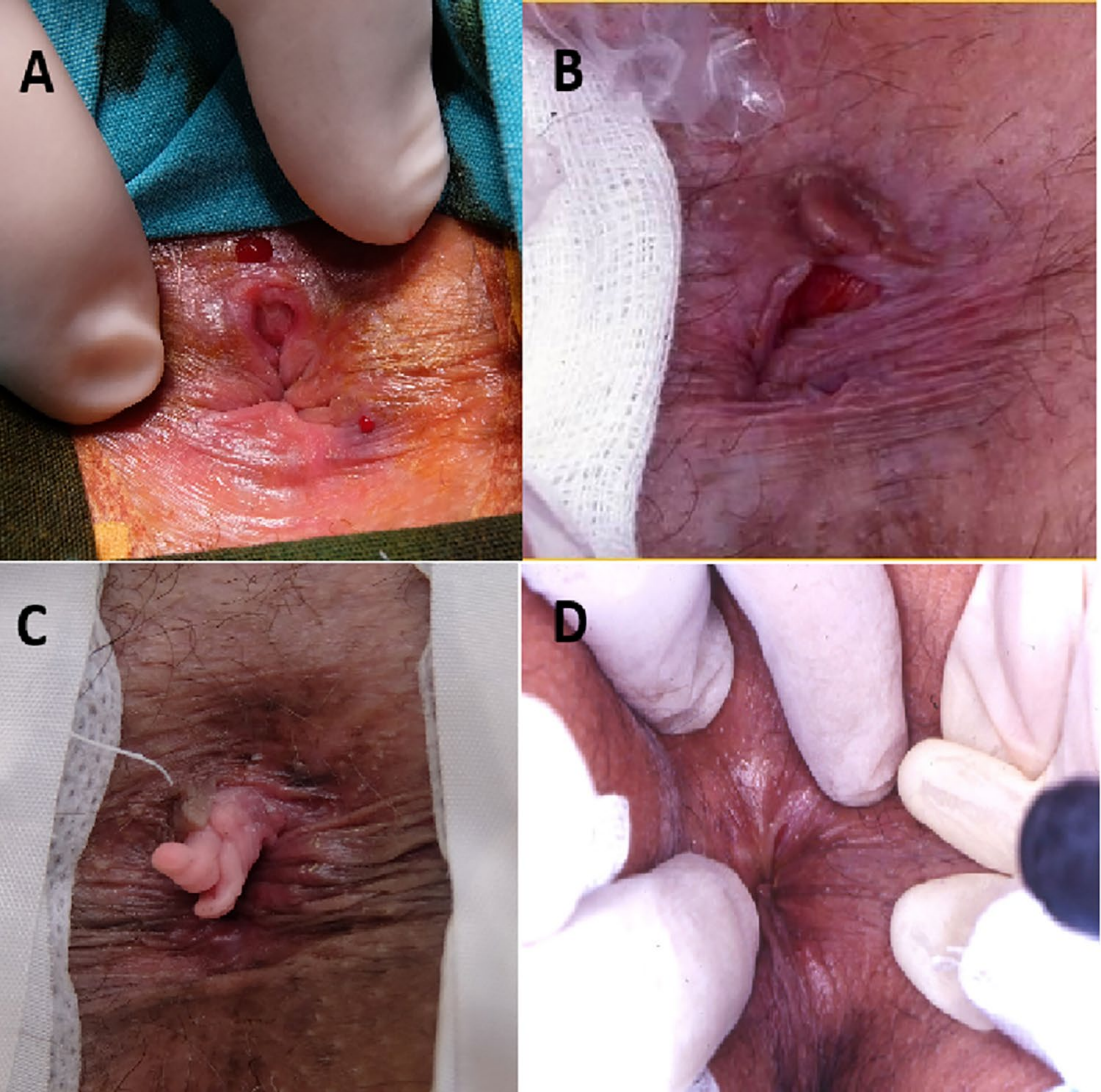
Sentinel pile in chronic anal fissure: representative clinical images. A typical CAF with the horizontal fibers of the IAS visible in its base. The skin inflammation and edema around the fissure are not regarded as SP (**A**); a typical CAF with prominent SP (**B**); an extreme, fibrotic, horny SP (**C**); and a rather inactive, chronic fissure—an intermediate lesion without overt inflammatory findings and visible IAS (incomplete healing) (**D**)

**Fig. 2:**
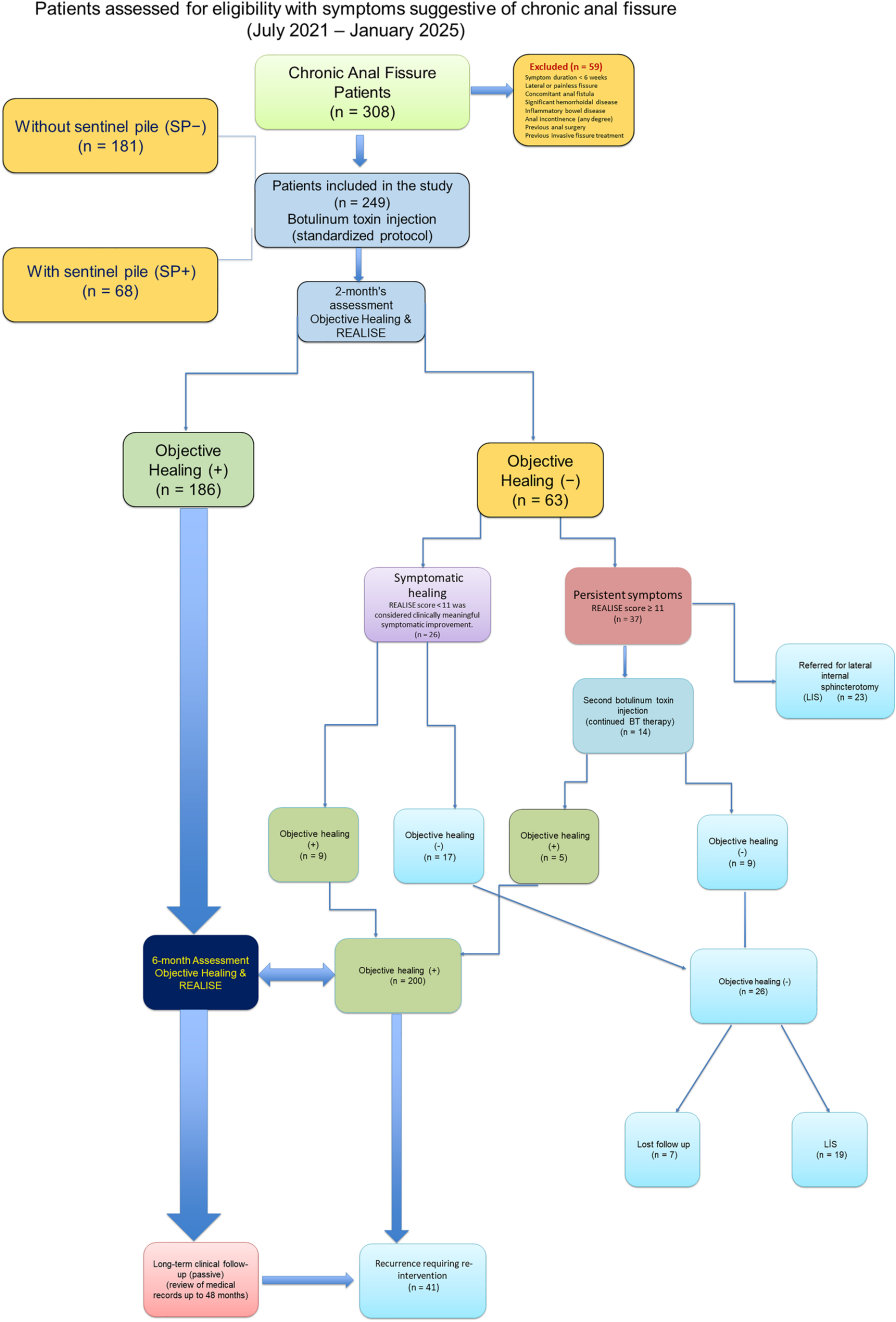
Study flowchart illustrating patient selection, treatment pathways, and clinical outcomes

Patients were included in the analyses if symptoms attributable to CAF had persisted for at least 6 weeks. Only those patients who had severe, chronic fissure with the horizontal fibers of the IAS visible in its base were chosen (confirmed by two proctologists), (Fig. 1A). The patients included in our study uniformly had increased anal/sphincter tonus, and it was even difficult to fully examine the anal canal because of the pain, hypersensitivity, and increased tonus. The presence of a clinically prominent sentinel pile was recorded at baseline examination (Fig. 1B, C). SP was defined as a fibrotic, well-demarcated skin tag, clearly distinguishable from inflammatory edema, with a maximal diameter of ≥ 5 mm on clinical examination. Patients with laterally located or painless fissures were not included. In our clinical practice, BT injection is not used as first-line therapy for chronic anal fissure, and only patients who had received and failed conservative/medical treatment were included in the study [9, 18]. Other exclusion criteria were concurrent fistula or significant hemorrhoidal disease, inflammatory bowel disease, anal incontinence of any degree, slow-transit-time constipation, diabetes or other metabolic/endocrine disorders, alcoholism, drug abuse, anoreceptive intercourse, or previous anal surgery. Patients were enrolled consecutively between July 2021 and January 2025 and their data were recorded on standardized forms, which were then analyzed retrospectively. All patients provided written informed consent. The study was approved by the Ethics Committee of Memorial Ankara Hospital (approval no. MEM-ANK-25-4). The study was not pre-registered, as it was designed as a retrospective analysis; however, all predefined methodological steps were strictly adhered to, and no deviations from the planned protocol occurred. Only those patients who confirmed to participate on the day of Botox injection at 7th-day control were included in further analyses. The reporting of this study follows the Strengthening the Reporting of Observational Studies in Epidemiology (STROBE) guidelines to ensure transparency and completeness. A detailed flowchart illustrating patient enrollment, exclusions, treatment pathways, and follow-up is shown in Fig. 2.

### Botulinum toxin injection

Type A Botulinum toxin (Botox^®^, Allergan, Irvine, CA) was diluted in saline to 50 IU/ml. Under intravenous sedation and in lateral decubitus position, the perianal area was cleaned with 10% povidone iodine and the IAS was palpated and fixed between the index finger and thumb of the left hand. A total of 1 IU/kg of Botox^®^ was injected in two equal volumes on each side of the anterior midline with a 25-gauge needle, targeting the center of the IAS halfway between the anal verge and dentate line.

No local anesthetics or any other medications were used before, during, or after the procedure. Following BT injection, institutional brochures were given to correct general causes of constipation, such as irregular meals, poor toilet facilities, and lack of exercise. Whenever possible, warm shower application after each bowel movement was also encouraged. They were free to return to daily activities once sedation had worn off.

### Follow-up and evaluation of healing

The patients were examined on postinjection day 7, and then at 2 and 6 months. Telephone contacts at 12 months and then on a yearly basis were part of the routine follow-up of the proctology unit. The patients were encouraged to come immediately and not to wait for the next interview if they developed symptoms. Objective fissure status was evaluated by the same blinded investigator, who was unaware of cumulative results or prior records. One of the three descriptions was noted: (i) deep, edematous, and/or easily bleeding ulcer, with IAS visible (by definition a CAF or nonhealing); (ii) a rather inactive, chronic fissure—an intermediate lesion—without overt inflammatory findings and visible IAS (incomplete healing), (Fig. 1D); or (iii) a contracted and completely epithelized scar or no signs of fissure (considered as objective healing). For patients with dual fissures, the worse lesion determined the outcome. The lack of objective healing response 2 months after BT injection was regarded as early failure, and such patients were suggested to undergo a second BT or sphincterotomy (data not shown).

In addition to objective assessment, symptom severity was scored using the REALISE score, which covers five items: pain severity (visual analog scale [VAS]), pain duration, analgesic requirement, bleeding, and impact on quality of life (QoL) [17]. Scores range from 4 to 30. Assessments were performed immediately before BT injection and at 6 months or at 2 months for early failures. The background etiology of hypertonicity was not an attempted and measured parameter within the limits of the present study design although, clinically, this was apparent in all patients.

### Statistical analysis

The Shapiro–Wilk test was used to assess normality of continuous variables. Normally distributed data were presented as mean ± standard deviation (SD) and compared with the independent-samples *t*-test. Non-normally distributed variables were expressed as median (interquartile range [IQR], min–max) and compared with the Mann–Whitney *U*-test or Wilcoxon signed-rank test (pre- versus post-treatment). Pearson’s chi-squared test was used for categorical variables; Fisher’s exact test was used for 2 × 2 tables when expected cell frequencies were < 5. The effect of SP presence on changes in symptom scores after BT was analyzed with a two-way mixed-effects analysis of variance (ANOVA), using group (SP+ versus SP−) as the between-subject factor and time (baseline versus post-treatment) as the within-subject factor. Within-group changes were additionally tested with the Mann–Whitney *U*-test for robustness. To account for multiple testing, Bonferroni correction was applied. Variables with *p* < 0.20 in the univariate analysis, along with other clinically important variables, were then considered in a multiple logistic regression model to determine independent risk factors. Adjusted odds ratios (ORs) and corresponding 95% confidence intervals (CIs) were reported for each predictor. All *p*-values were two-tailed, and *p* < 0.05 was considered significant. The analyses were performed using IBM SPSS Statistics, version 26.0 (IBM Corp., Armonk, NY, USA).

## Results

Of the initial 308 patients with CAF, 249 patients who underwent BT treatment between July 2021 and January 2025 met the inclusion criteria, confirmed follow-up, and were analyzed. The median age was 41 years (range 19–82 years) with 78 females (31.3%) and 171 males (68.7%). Patients were followed for at least 6 months (range 6–48 months, median 34 months). In total, 193 patients harbored posterior fissures (77.5%), 16 anterior fissures (6.4%), and 40 had dual fissures (16.1%). Of the 249 patients, 68 presented with prominent SP (27.3%).

The overall objective healing rate following a single BT injection was 74.7% (186 patients) at 2 months. No significant difference in sex distribution was found among the patients who exhibited positive objective healing outcomes; that is, male or female patients healed in similar percentages (*p* = 0.876). Of the 63 patients with objective lack of healing, 14 underwent a second BT injection, and 23 underwent sphincterotomy. In addition, 26 patients with incomplete objective healing but with symptomatic remission were also included in the 6-month analyses. With a second BT injection in 14 initial failures, the overall healing rate reached 80.3% at 6 months. Minor complications were observed in 13 patients (5.2%), who developed small, sometimes thrombosed, hemorrhoids that resolved spontaneously. A total of 15 patients (6%) reported transient gas incontinence.

The median pre-treatment REALISE score was 18 (IQR 6), which decreased significantly to 6 (IQR 3) at 6 months post treatment (*p* < 0.001). All five individual items of the REALISE score—pain severity (VAS), pain duration, analgesic use, bleeding, and QoL impact—showed significant improvements (*p* < 0.05 for all), (Table 1).

**Table 1:** Demographics and some clinical features of patients with chronic anal fissure with or without sentinel piles treated with botulinum toxin

Variable	Overall (*n* = 249)	SP− (*n* = 181)	SP+ (*n* = 68)	*p* value
Age, years—median (range) (IQR)	41 (19–82)	41.0(15)	39.5 (13)	NS (0.545^Ω)^
*Sex—n (%)*				
Female	78 (31.3%)	48 (26.5%)	30 (44.1%)	0.009^*^
Male	171 (68.7%)	133 (73.4%)	38 (55.9%)	
Symptom duration, weeks —median	–	12	16	NS 0.247^Ω^
Objective healing after first BT injection—*n* (%)	186 (74.7%)	146 (80.7%)	40 (58.8%)	< 0.001^*^
Objective healing after second BT injection—*n* (%)	200 (80.3%)	153 (84.5%)	47 (69.1%)	0.011^*^
Pre-treatment REALISE score—median (IQR)	18 (6)	18 (7)	18 (6)	NS 0.871^†^
Post-treatment REALISE score—median (IQR)	6 (3)	5 (3)	7 (4)	**< 0.001**^**†**^
Post-treatment Q1 score	1 (2)	1 (2)	2 (1)	**< 0.001**^**Ω**^
Post-treatment Q2 score	1 (0)	1 (0)	1 (1)	**0.003**^**Ω**^
Post-treatment Q3 score	1 (0)	1 (0)	1 (1)	**0.002**^**Ω**^
Post-treatment Q4 score	1 (1)	1 (0)	1 (1)	**0.039**^**Ω**^
Post-treatment Q5 score	1 (1)	1 (1)	1 (1)	**0.017**^**Ω**^
Recurrence during follow-up *n* = 226 (%)	41 (18.1%)	27 (15.7%)	14 (25.9%)	NS 0.106^¥^

Data are presented as median (interquartile range) or number (%). *p* values refer to comparisons between with sentinel pile (SP+) and without sentinel pile (SP−) groups. Bold values indicate statistically significant results (p<0.05). ^†^Wilcoxon signed-rank test, ^¥^Fisher’s exact test, ^*^chi-squared test, ^Ω^Mann–Whitney *U*-test

*BT* botulinum toxin, *IQR* interquartile range, *NS* not significant

When stratified by the SP status, age distribution did not differ significantly (*p* = 0.545), but SP was more common in females than in males (38.5% versus 22.5%; *p* = 0.009). The duration of symptoms was slightly longer in SP+ patients compared with SP−(16 versus 12 weeks), but this difference was not statistically significant (*p* = 0.247). Baseline total REALISE scores were comparable between groups [SP+ : 18.0 (6) versus SP−: 18.0 (7); *p* = 0.871], as were the sub-scores (*p* > 0.05 for all).

**Fig. 3:**
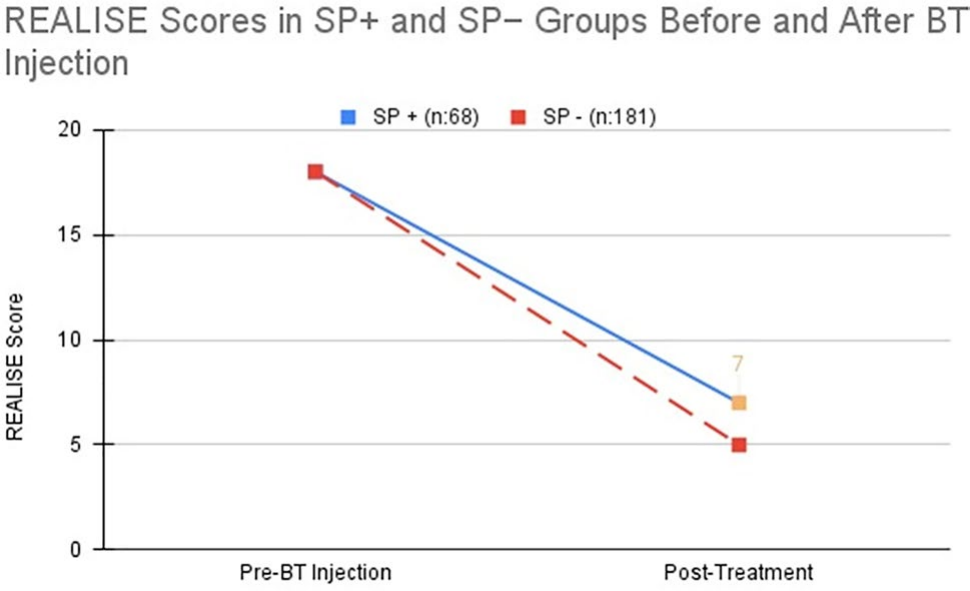
Symptom scores (REALISE) in patients with sentinel pile (SP+) or without sentinel pile (SP−) before and 6 months after BT injection. Repeated-measures ANOVA, F (1247) = 7.061, *p* = 0.008, confirmed by Mann–Whitney *U*-test,* p* = 0.005

Objective healing after a single BT injection was 80.7% (146 of 181) in SP− patients and 58.8% (40 of 68) in SP+ patients, indicating to a statistically significant difference (*p* = 0.001). With a second BT injection, healing increased to 84.5% in SP− and 69.1% in SP+ patients, but the difference remained significant (*p* = 0.011). Symptom scores improved significantly in both groups [SP−: 18 (7) to 5 (3), *p* < 0.001; SP+ :18 (6) to 7 (4), *p* < 0.001]. However, post-treatment scores were higher in the SP+ group compared with SP− (*p* < 0.001), confirming reduced responsiveness in SP+ patients. A two-way repeated-measures ANOVA showed a significant main effect of SP presence on symptom improvement (F [1247] = 7.061, *p* = 0.008), confirmed with the Mann–Whitney *U*-test (*p* = 0.005) for robustness (Fig. 3).

In multivariable logistic regression analysis, sentinel pile presence remained independently associated with a lower likelihood of healing (OR 0.258, 95% CI 0.130–0.512, *p* < 0.001). Higher pre-treatment REALISE scores were also independently predictive of reduced healing probability (OR 0.827 per point, 95% CI 0.766–0.893, *p* < 0.001). In contrast, age and sex were not significant predictors (Table 2).

**Table 2:** Multivariable logistic regression for predictors of healing after BT injection

Variable (*n* = 249)	OR (Exp(B))	95% CI for OR	*p*-value
Age (years)	0.996	0.969–1.023	0.768
Sex (male versus female)	0.638	0.312–1.306	0.219
Pre-treatment REALISE score (per point)	0.827	0.766–0.893	**< 0.001**
Sentinel pile (+ versus −)	0.258	0.130–0.512	**< 0.001**

Model fit: omnibus χ^2^ = 39.64, *p* < 0.001; Nagelkerke *R*^2^ = 0.217; Hosmer–Lemeshow *p* = 0.689; overall accuracy = 77.9%. Bold values indicate statistically significant results (p<0.05)

During the median follow-up of 34 months, recurrence or loss to follow-up (considered treatment failure) occurred in 41 patients (18.1%). Recurrence was higher in the SP+ group (14 cases, 25.9%) compared with the SP− group (27 cases, 15.7%), although this did not reach statistical significance (*p* = 0.106).

## Discussion

The results of the present study have confirmed that BT injection provides considerably high healing rates in patients with CAF. We had previously demonstrated that BT injection exhibited a significant healing rate of 73.8% following a single injection, with an overall success rate of 86.9% upon potential retreatment. This intervention not only resulted in a lower incidence of complications compared with sphincterotomy but also facilitated a quicker return to daily activities although the early and late healing rates were lower than those of the sphincterotomy group [11]. In the current study, we observed similar outcomes, with an overall objective healing rate of 74.7% after a single injection and 80.3% with an additional injection in selected failure. Moreover, fissure-related symptoms improved significantly. We cannot contradict that different dose regimens or applications might result in different clinical healing rates.

The skin tag, sentinel pile (SP) was first described by Sir Alfred Brodie in the 1950s, highlighting its significance as a clinical indicator associated with anal fissures [19]. Although sentinel pile is commonly associated with chronic anal fissure, its absence does not preclude the diagnosis of chronicity in patients with prolonged symptoms and characteristic morphological findings [20]. Only 27.3% of patients harbored a prominent sentinel pile. However, the reason for the presence or absence of a SP is not well known. It is speculated to be a skin sequel resulting from the repeated attacks and remissions of the fissure. The present study provides information on sentinel pile, a topic that remains insufficiently explored in literature. In total, 27% of the patients harbored a significant SP. Female patients developed an SP more often than male patients (38.5% versus 22.5%; *p* = 0.009). Currently, insufficient data exist to explain why SP is present only in some patients or why it appears more prevalent in women. The symptom duration of patients with a SP was not significantly longer than that of the patients without SP. This finding challenges the assumption that disease duration alone is the primary determinant of local tissue changes leading to sentinel pile formation. Although chronic inflammatory and fibrotic processes may still play a role, the development of a sentinel pile does not appear to correlate with baseline symptom severity, as reflected by comparable pre-treatment REALISE scores. Despite the higher prevalence of SP in women, healing rates were similar between sexes. Nevertheless, the retrospective and single-center design of present study limits the generalizability of these observations, and larger datasets may yield different statistical outcomes. Interpretations derived from the present findings are subject to several limitations. The retrospective design with inherent risk of bias, utilizing telephone follow-up for long-term evaluation, potential lack of comparability between the groups at baseline and/or clinical ambiguity between sentinel pile versus surrounding inflammatory edema put shade on causal conclusions. Furthermore, symptom duration was based on patient-reported history documented in clinical records rather than prospectively collected data, which may introduce recall bias and should be considered a further limitation of the study. In addition, analyzing patients who required a second BT injection together with single-injection responders, as part of a pragmatic treatment algorithm, may limit direct comparability between these subgroups and should be considered a methodological limitation. Nevertheless, the present study is one of the first to address a well-known but insufficiently explored topic, and it might inspire further studies to define the pathogenesis and clinical impact of the sentinel pile.

The more clinically relevant finding of this study is the impact of SP presence on treatment outcomes. Although based on non-randomized retrospective data, the present detailed evaluation of well-documented features in patients with CAF with or without SP might provide new insights for clinical decision-making. The REALISE score was used to assess symptom severity in a multidimensional manner, as previously described. The present findings showed that while symptom severity decreased significantly in both SP+ and SP− groups, post-treatment REALISE scores remained higher in the SP+ group. Thus, SP+ patients were less responsive both objectively and subjectively to BT treatment, indicating that the presence of SP may negatively influence healing. Besides, the persistence of some disturbance after the treatment can derive directly from the permanence of the pile itself. Symptomatic improvement without complete epithelial healing was interpreted as a distinct clinical outcome rather than equivalent to objective healing. The presence of SP and higher pre-treatment REALISE scores were independently associated with lower objective healing rates after BT injection, warranting further investigation.

Regarding the healing rates following BT injection, the objective healing rate was also significantly lower in patients with SP. Scarce previous studies have suggested that association with an SP is a predictor of failure of treatment with glyceryl trinitrate (GTN) [16, 21] or recurrence following BT [22]. Nevertheless, about 60% of patients with SP still healed, determined by strict objective criteria, although this healing rate was higher in SP− patients. Unlike the deduction of Arroyo and coworkers, the recurrence rate of 25.9% in the SP+ group and 15.7% in the SP− group did not differ significantly in the present study (*p* = 0.106).

In conclusion, the presence of SP might affect the clinical presentation and BT treatment of patients with CAF. The results of the present patient cohort suggest that:

- *(i)* Approximately one-quarter of patients with CAF present with prominent SP.
- *(ii)* SP is more common in women than in men.
- *(iii)* Symptom duration is not significantly different between SP+ and SP− patients.
- *(iv)* Objective healing rates and symptom improvement after BT injection are lower in SP+ patients.
- *(v)* Recurrence rates are numerically higher in SP+ patients but without statistical significance.

Nevertheless, even with the SP, a considerable proportion of patients with CAF heal after BT treatment, and their symptoms are generally relieved. However, neither objective healing nor symptomatic improvement is as good as in patients without SP. It is worthwhile to remember that these patients usually think that it is the SP that causes pain and other problems. Although the presence of an SP is not a contraindication for BT treatment, further data may be required before expert groups or guideline committees can evaluate whether treatment strategies should be refined for specific patient subgroups. At minimum, patients should be informed that the presence of SP may negatively influence clinical healing following BT treatment. The present study might inspire further data and analyses regarding CAF with or without SP, and this should ideally be provided by prospective, multicenter, randomized controlled trials.

## Data Availability

The datasets generated and/or analyzed during the current study are not publicly available owing to patient confidentiality but are available from the corresponding author on reasonable request.
